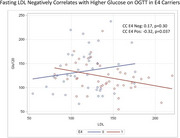# Impact of APOE E4 on Glucose Tolerance and Lipid Profiles in Older Adults

**DOI:** 10.1002/alz70860_098069

**Published:** 2025-12-23

**Authors:** Pooja Thorali, Kristen M Farris, Jasroop K Miglani, Angela J Hanson

**Affiliations:** ^1^ University of Washington School of Medicine, Seattle, WA, USA

## Abstract

**Background:**

Type 2 diabetes and elevated LDL cholesterol are reversible risk factors for Alzheimer's disease (AD), and the E4 allele of the APOE 4 gene is a genetic risk for AD. We have previously published that E4 status influences results on glucose tolerance test in those with early AD, and this project examines the relationship between glucose, lipids and APOE in healthy older adults.

**Method:**

183 older adults (mean age 86 ± SD 7.4, 54.6% female) underwent a blood draw for fasting blood sugar, hemoglobin A1c and fasting lipids as a part of screening for research studies, as well as vitals and APOE genotyping. A subset underwent an oral glucose tolerance test (OGTT) with glucose measured at 0 and 2 hours. Statistics performed in SAS.

**Result:**

After adjusting for BMI, E4 carriers had lower average fasting glucose (mean ± SEM 90.3 ± 1) compared to non‐carriers (94.1 ± 1, *p* = 0.0112) and a trend toward lower A1C (*p* = 0.0538). When examined by all APOE genotypes, E4 homozygotes had the lowest glucose (87.7 ± 2.6) compared to E22 and E23 carriers (96.2 ± 2.2, E2 vs E44 *p* = 0.0137). In contrast, E4 carriers had higher LDL than non‐carriers (121 ± 3 vs 131 ± 3, *p* = 0.0293), and E44 group had the highest (143 ± 8) and E2 group the lowest LDL (97 ± 7, *p* = <0.001) when comparing by genotype. A subset underwent OGTT, and E44 group had the lowest average rise in glucose at 2 hours (89.7 ± 16) compared to E2 group (133 ± 12.6, *p* = 0.0352). Correlations between risk factors revealed that several risk factors positively correlated with 2 hour glucose in non‐carriers, whereas in E4 carriers, fasting LDL negatively correlated with 2 hour glucose (Figure).

**Conclusion:**

Despite the presence of dyslipidemia, E4 carriers demonstrated a statistically and clinically lower blood glucose both fasting and post‐OGTT and appear to have a different relationship with plasma lipids and prandial glucose. These findings may have implications for AD prevention, including using diabetes medications to treat AD.